# Superiority of Microencapsulated Essential Oils Compared With Common Essential Oils and Antibiotics: Effects on the Intestinal Health and Gut Microbiota of Weaning Piglet

**DOI:** 10.3389/fnut.2021.808106

**Published:** 2022-01-12

**Authors:** Kaibin Mo, Jing Li, Fenfen Liu, Ying Xu, Xianhui Huang, Hengjia Ni

**Affiliations:** ^1^Guangdong Key Laboratory for Veterinary Drug Development and Safety Evaluation, College of Veterinary Medicine, South China Agricultural University, Guangzhou, China; ^2^National Engineering Laboratory for Pollution Control and Waste Utilization in Livestock and Poultry Production, Hunan Province Key Laboratory of Animal Nutritional Physiology and Metabolic Process, Key Laboratory of Agro-ecological Processes in Subtropical Region, Institute of Subtropical Agriculture, Chinese Academy of Sciences, Changsha, China

**Keywords:** weaning piglets, essential oils, microencapsulation, intestinal health, microbiota

## Abstract

Essential oils (EOs) have long been considered an alternative to antibiotics in the breeding industry. However, they are unstable and often present unpleasant odors, which hampers their application. Microencapsulation can protect the active gradients from oxidation and allow them to diffuse slowly in the gastrointestinal tract. The purpose of this study was to investigate the effect of microencapsulation technology on the biological function of EOs and the possibility of using microencapsulate EOs (MEEOs) as an alternative to antibiotics in weaning piglets. First, we prepared MEEOs and common EOs both containing 2% thymol, 5% carvacrol and 3% cinnamaldehyde (w/w/w). Then, a total of 48 weaning piglets were randomly allotted to six dietary treatments: (1) basal diet; (2) 75 mg/kg chlortetracycline; (3) 100 mg/kg common EOs; (4) 500 mg/kg common EOs; (5) 100 mg/kg MEEOs; and (6) 500 mg/kg MEEO. The trial lasted 28 days. The results showed that piglets in the 100 mg/kg MEEOs group had the lowest diarrhea index during days 15–28 (*P* < 0.05). In addition, 100 mg/kg MEEOs significantly alleviated intestinal oxidative stress and inflammation (*P* < 0.05), whereas 500 mg/kg common EOs caused intestinal oxidative stress (*P* < 0.05) and may lead to intestinal damage through activation of inflammatory cytokine response. MEEOs (100 mg/kg) significantly reduced the ratio of the relative abundance of potential pathogenic and beneficial bacteria in the cecum and colon (*P* < 0.05), thus contributing to the maintenance of intestinal health. On the other hand, chlortetracycline caused an increase in the ratio of the relative abundance of potential pathogenic and beneficial bacteria in the colon (*P* < 0.05), which could potentially have adverse effects on the intestine. The addition of a high dose of MEEOs may have adverse effects on the intestine and may lead to diarrhea by increasing the level of colonic acetic acid (*P* < 0.05). Collectively, the results suggest that microencapsulation technology significantly promotes the positive effect of EOs on the intestinal health of weaning piglets and reduces the adverse effect of EOs, and 100 mg/kg MEEOs are recommended as a health promoter in piglets during the weaning period.

## Introduction

Weaning piglets often suffer severe diarrhea due to weak immunity, immature gastrointestinal tracts and sudden changes in dietary structure. Weaning stress can reduce the growth performance of piglets and even cause death, which is a vital cause of financial losses to the pig industry (1). In the last century, antibiotic growth promoters (AGPs) were widely used in weaning piglets due to their ability to suppress pathogens and modulate the immune system (2). However, the abuse of AGPs can cause intestinal dysbiosis in piglets; even worse, it has brought about widespread bacterial antibiotic resistance (3). Therefore, AGPs have been banned in many countries to prevent the further spread of bacterial antibiotic resistance (4). The development of antibiotic alternatives has become an urgent issue in the swine industry.

Essential oils (EOs) are compounds with biological activity extracted from plants, and they have long been considered an alternative to antibiotics and used for treating various diseases, such as acute/chronic gastroenteritis, heat stress, reproductive system dysfunction and even cancer (5). Two major groups of compounds are commonly used: terpenes (e.g., thymol, limonene and carvacrol) and phenylpropanoids (e.g., cinnamaldehyde, safrole and eugenol) (6). Thymol, carvacrol and cinnamaldehyde are three of the most widely used and well-researched EOs and exhibit strong antimicrobial activities. The delocalized electron system in cinnamaldehyde and the phenolic hydroxyl groups in carvacrol and thymol are key to the antibacterial activities (7). *In vitro* trials have confirmed that an EO mixture containing thymol, eugenol, and carvacrol has high antimicrobial activity against *Salmonella, Staphylococcus aureus* and *Escherichia coli* (8).

In the swine industry, thymol, carvacrol and cinnamaldehyde are often used to improve growth performance and treat postweaning diarrhea due to their antibacterial, anti-inflammatory and antioxidant properties (6). Many studies have found that the combination of different EO compounds may have a better performance than each compound taken individually. Wei found that dietary supplementation with a mixture of carvacrol and thymol decreased intestinal oxidative stress in weaning piglets (9). Li reported that diets supplemented with thymol and cinnamaldehyde increased the ratio of *Lactobacilli* to *E. coli* in the colon, which had a positive effect on intestinal health in weaning piglets (10). However, most EOs, including carvacrol and cinnamaldehyde, are unstable (rapid evaporation and degradation) in the presence of air, light, moisture and high temperatures. In addition, EOs often present an unpleasant odor. These factors greatly hamper the application of EOs in the breeding industry, which require the ingredients to be odor free and possess good chemical stability (6). Thus, it is important to find a way to improve the stability of EO products.

Encapsulation, defined as the process by which microparticles are encapsulated within a coating (or embedded) in a homogeneous or heterogeneous matrix, is thought to enhance the stability of core materials (11). Currently, vitamins, peptides, and probiotics are often encapsulated in lipid-based matrix microspheres to improve their bioavailability in the feed industry (12). This food processing technology has also been applied to EO processing (13). Solid microencapsulated essential oils (MEEOs) do not possess unpleasant odors and can tolerate high temperatures during the feed pelleting process, which makes them more practical (14).

It is speculated that the biological effects of microencapsulated EOs and common EOs are different due to microencapsulation technology. However, few studies have evaluated the effect of microencapsulation technology on the biological function of EOs and the effect of EOs and MEEOs as an alternative to AGPs for weaning stress in piglets. Therefore, in this study, we chose thymol, carvacrol, and cinnamaldehyde to prepare a MEEO and compared the functional differences between the MEEOs and common EOs and antibiotics in relieving diarrhea and regulating the intestinal microbiota in weanling piglets.

## Materials and Methods

### Preparation of MEEO

Thymol (> 95% purity), carvacrol (> 95% purity), and cinnamaldehyde (> 97% purity) were supplied by Jinhe Biotechnology Company (Hohhot, China). MEEO (containing 2% thymol, 5% carvacrol and 3% cinnamaldehyde) was prepared according to a method described by Hoyos-Leyva et al. (15) and Marefati et al. (16). Briefly, sucrose fatty acid ester (163.0 kg), amylum (70.0 kg) and sodium aluminosilicate (27.5 kg) were added into a preheated (90°C) emulsion tank with a shear-driven emulsification system (HR-2L, HUXI Industrial Co., Shanghai, China) and stirred thoroughly to stimulate the liquid-solid transition. Then, the emulsion tank was set to 60°C and carvacrol (15.8 kg), cinnamaldehyde (9.5 kg) and thymol (6.2 kg) were added to the emulsion tank and mixed well, and the shear-driven emulsification system enabled the ingredients to be completely emulsified. The completely emulsified materials were applied to a low-speed spray granulator (YC03, Ya Cheng Instrument & Equipment Co., Shanghai, China) for granulation. The particles were passed through a 40–60 mesh sieve and stored in an airtight container. The particle size, microencapsulation yield and the thermal stability were analyzed, and the data are shown in the Supplementary Material (Supplementary Data 1.1–1.3).

### Animals and Treatments

Forty-eight healthy weaned piglets (Landrace × Large White, 21 days old, 6.26 ± 0.43 kg B.W.) were randomly assigned to six dietary treatments with 8 piglets per treatment: control group (Con) received the basal diet; CTC group received the basal diet + 75 mg/kg chlortetracycline; low dose EO group (LEO) received the basal diet + 100 mg/kg common EOs; high dose EO group (HEO) received the basal diet + 500 mg/kg common EOs; low dose MEEO group (LMEEO) received the basal diet +100 mg/kg MEEOs; high dose MEEO group (HMEEO) received the basal diet + 500 mg/kg MEEOs. The common EOs were prepared using the exact same ingredients as MEEOs. Each piglet was housed in an individual metabolism cage (1.5 m length × 0.8 m width) and provided free access to feed and water. The trial lasted for 28 days. The nutritional levels of the diets met the National Research Council (2012) nutrient recommendation, and the composition of the basal diet is shown in Supplementary Table 2.

### Growth Performance and Diarrhea Index

The body weights (BWs) of the piglets were measured after 12 h of fasting on the mornings of Day 1, Day 14 and Day 28. Feed intake was recorded daily for calculating average daily feed intake (ADFI), average daily weight gain (ADG) and feed conversion ratio (FCR). The status of feces was observed by an observer at the same time of day throughout the experiment, and the diarrhea index was analyzed according to a previous study (17).

### Sample Collection

After being fasted for 12 h, the piglets were anesthetized by the injection of sodium pentobarbital (90 mg/kg BW). Blood samples were collected from the anterior vena cava, and serum samples were prepared by centrifugation at 3,500 g for 15 min at 4°C. The segments of the duodenum, jejunum, and ileum, as well as the digesta from the cecum and colon, were quickly collected and stored at −80°C for further analysis.

### Serum Biochemical Parameter Analysis

Serum concentrations of total protein (TP), albumin (ALB), lactic acid (LACT), alkaline phosphatase (ALP), and diamine oxidase (DAO) were determined using commercial kits (F. Hoffmann-La Roche Ltd, Basel, Switzerland) and a Roche automatic biochemical analyzer (Cobas c311, F. Hoffmann-La Roche Ltd, Basel, Switzerland) in accordance with the manufacturer's instructions.

### Analysis of Antioxidant Capacity in Small Intestine

The total antioxidant capacity (T-AOC), malondialdehyde (MDA) content, and superoxide dismutase (SOD), catalase (CAT) and glutathione peroxidase (GSH-Px) enzyme activities in the duodenum, jejunum and ileum were determined using spectrophotometric kits (Jiancheng Bioengineering Institute, Nanjing, China) in accordance with the manufacturer's instructions.

### Real Time-Quantitative PCR

The expression of the following inflammation-related genes in the duodenum, jejunum and ileum was determined by Real time-quantitative PCR: tumor necrosis factor-α (*TNF-*α), interleukin (*IL*)-*1*β, *IL*-*4, IL*-*8*, Toll-like receptor (*TLR*) *4* and *TLR8*. The primers used in this study are listed in Supplementary Table 3. Total RNA was extracted using TRIzol reagent (Invitrogen, Carlsbad, CA, USA), and the concentration was determined using a NanoDrop 2000 spectrophotometer (Thermo Scientific, Wilmington, DE, USA). cDNA was synthesized using the PrimeScriptTM RT reagent kit with a genomic DNA eraser (Takara Bio, Japan). β*-Actin* was used as the internal control to normalize the target gene transcript levels. qPCR was performed in a 10 μl reaction volume using a SYBR Premix Ex Taq kit (Takara, Japan). PCR amplification was performed according to a previous study (18).

### Determination of Short-Chain Fatty Acids (SCFAs)

The amount of colonic SCFAs (acetate, propionate, butyrate and valerate) was determined by UPLC–MS/MS according to a previous study (19). Briefly, 50 mg of homogeneous colonic digesta was mixed with 1 mL extraction (methanol: ddH_2_O = 1: 1), and the mixture was vortex extracted for 30 min, followed by centrifugation for 10 min (12,000 g/min). Then, 50 μl of internal standard and 50 μl of derivatizing reagent were added to 50 μl of supernatant and mixed well to allow the samples to be derivatized at room temperature for 30 min. After derivatization, 50 μl of protectant and 250 μl of water were added to the mixture and vortexed for 10 s. Finally, the mixture was centrifuged for 10 min (12,000 g/min), and 500 μl supernatant was taken for UPLC–MS/MS analysis. The UPLC–MS/MS method is described in Supplementary Tables 4, 5.

### Bacterial DNA Extraction and 16S rDNA Gene Sequencing

Microbial genomic DNA from cecal and colonic digesta was extracted using a QIAamp DNA Stool Mini Kit (Qiagen, Hilden, Germany). The V3–V4 region of the bacterial 16S rDNA was amplified with primers 341F (5′- CCTAYGGGRBGCASCAG-3′) and 806R (5′-GGACTACHVGGGTWTCTAAT-3′) using the following program: 98 °C for 2 min; 30 cycles of 10 s at 98 °C, 30 s at 50 °C, 30 s at 72 °C; and a final extension at 72 °C for 8 min. The PCR products were purified with the QIAquick PCR purification kit (Qiagen, Hilden, Germany). The purified amplicons were prepared using the TruSeq DNA PCR-Free Library Preparation Kit for Illumina (New England Biolabs, USA). Sequencing was performed on the Illumina HiSeq platform (Novogene Bioinformatics Technology Co., Ltd, Beijing, China).

### Statistical Analysis

One-way ANOVA with Duncan's multiple comparison test (SPSS 26.0 software) was used to analyze significant differences between groups. All data are presented as the mean and mean ± SEM. *P* < 0.05 were considered statistically significant.

## Results

### Growth Performance

The growth performance and diarrhea index results are summarized in Table 1. Dietary supplementation with 500 mg/kg MEEOs significantly (*P* < 0.05) increased the ADG of piglets from Days 1 to 14 compared with the Con, CTC and HEO groups. Moreover, the ADFI of piglets tended to increase with increasing MEEOs addition from Days 1 to 14. Piglets in the LMEEO group had less diarrhea index than those in the HEO group (*P* < 0.05), and piglets in the CTC, LEO and LMEEO groups had similar diarrhea indices from Days 15 to 28.

**Table 1:** Effect of common EOs and MEEOs on the growth performance of weaning piglets.

**Item**	**Groups^1^**						**SEM^2^**	***P*-Value**
	**Con**	**CTC**	**LEO**	**HEO**	**LMEEO**	**HMEEO**		
**Day 1–14**								
ADG (g)	104.29^b^	108.10^b^	130.24^ab^	104.05^b^	131.55^ab^	162.74^a^	2.78	0.023
ADFI (g)	259.88	257.5	297.14	249.11	275.48	321.67	18.01	0.088
FCR	2.53	2.43	2.39	2.60	2.19	2.03	0.19	0.381
Diarrhea index	1.13	1.00	1.15	1.20	0.81	1.23	0.19	0.658
**Day 15–28**								
ADG (g)	425.12	372.86	439.4	420.24	404.76	454.05	29.71	0.523
ADFI (g)	818.69	786.19	917.14	862.26	806.79	911.55	47.28	0.285
FCR	1.93	2.25	2.12	2.05	2.01	2.02	0.11	0.689
Diarrhea index	0.54^ab^	0.39^b^	0.31^b^	1.02^a^	0.30^b^	0.54^ab^	0.15	0.035
**Day 1–28**								
ADG (g)	264.7	240.48	284.82	262.14	268.15	308.39	18.15	0.214
ADFI (g)	539.29	521.85	607.14	555.68	541.13	616.61	28.96	0.152
FCR	2.05	2.26	2.17	2.14	2.04	2.00	0.11	0.696
Diarrhea index	0.83	0.70	0.73	1.11	0.55	0.88	0.15	0.250

*ADG, average daily gain; ADFI, average daily feed intake; FCR, feed conversion ratio*.

a, b *Different superscripts within a row indicate a significant difference (P <0.05)*.

1 *Con, basal diet; CTC, basal diet supplemented with 75 mg/kg chlortetracycline; LEO, basal diet supplemented with 100 mg/kg common EOs; HEO, basal diet supplemented with 500 mg/kg common EOs; LMEEO, basal diet supplemented with 100 mg/kg MEEOs; HMEEO, basal diet supplemented with 500 mg/kg MEEOs*.

2 *SEM means standard error of the means (n = 8)*.

### Serum Biochemical Parameter

The results of the serum biochemical parameters of piglets are shown in Table 2. Dietary supplementation with 100 mg/kg MEEOs significantly decreased serum DAO activity in piglets compared with the HEO and CTC diets (*P* < 0.05).

**Table 2:** Effect of common EOs and MEEOs on the serum biochemical parameters of weaning piglets.

**Item**	**Groups^1^**						**SEM^2^**	***P*-Value**
	**Con**	**CTC**	**LEO**	**HEO**	**LMEEO**	**HMEEO**		
TP (g/L)	48.59	47.29	48.53	47.29	47.86	47.73	1.45	0.975
ALB (g/L)	40.88	39.24	41.12	38.29	39.63	41.45	1.59	0.697
ALP (U/L)	275.00	285.71	310.00	261.88	272.75	276.83	19.38	0.658
DAO (U/L)	16.89^bc^	26.46^ab^	24.98^abc^	29.50^a^	13.40^c^	21.93^abc^	3.81	0.041
LACT (μmoL/L)	11.24	11.27	12.89	10.79	11.56	11.63	0.86	0.727

*TP, total protein; ALB, albumin; ALP, alkaline phosphatase; DAO, diamine oxidase; LACT, lactic acid*.

a, b, c *Different superscripts within a row indicate a significant difference (P <0.05)*.

1 *Con, basal diet; CTC, basal diet supplemented with 75 mg/kg chlortetracycline; LEO, basal diet supplemented with 100 mg/kg common EOs; HEO, basal diet supplemented with 500 mg/kg common EOs; LMEEO, basal diet supplemented with 100 mg/kg MEEOs; HMEEO, basal diet supplemented with 500 mg/kg MEEOs*.

2 *SEM means standard error of the means (n = 8)*.

### Intestinal Anti-Oxidative Capacity

As shown in Table 3, dietary supplementation with 500 mg/kg common EOs (HEO group) significantly increased duodenal MDA levels compared with other groups (*P* < 0.01). Piglets fed 100 mg/kg MEEOs showed higher duodenal T-AOC levels than those in the Con, CTC and LEO groups (*P* < 0.05). Piglets fed 500 mg/kg MEEOs had significantly increased jejunal GSH-Px activity compared with the Con and LMEEO groups (*P* < 0.05). The dietary treatments did not influence the antioxidative capacity in the ileum.

**Table 3:** Effect of common EOs and MEEOs on the intestinal anti-oxidative capacities in weaning piglets.

**Item**	**Groups^1^**						**SEM^2^**	***P*-Value**
	**Con**	**CTC**	**LEO**	**HEO**	**LMEEO**	**HMEEO**		
**Duodenum**								
T-AOC (mmol/gprot)	0.036^b^	0.035^b^	0.036^b^	0.042^ab^	0.048^a^	0.042^ab^	0.001	0.019
MDA (nmol/mL)	1.09^b^	0.91^b^	1.08^b^	1.90^a^	0.97^b^	1.17^b^	0.09	0.005
SOD (U/mgprot)	34.04	33.32	34	35.13	38.66	34.27	0.73	0.303
CAT (U/mgprot)	16.81	15.90	16.60	16.68	13.66	15.02	0.51	0.424
GSH-Px (U/mgprot)	19.79^bc^	18.75^c^	20.33^bc^	28.70^abc^	33.07^a^	30.24^ab^	1.59	0.016
**Jejunum**								
T-AOC (mmol/gprot)	0.034	0.036	0.033	0.038	0.039	0.054	0.002	0.113
MDA (nmol/mL)	0.15	0.14	0.11	0.20	0.20	0.21	0.12	0.070
SOD (U/mgprot)	40.92	41.44	41.50	40.26	39.30	38.19	0.85	0.858
CAT (U/mgprot)	18.50	18.31	15.68	16.52	15.00	13.45	0.67	0.181
GSH-Px (U/mgprot)	71.88^b^	82.42^ab^	81.93^ab^	81.38^ab^	69.59^b^	93.37^a^	2.36	0.041
**Ileum**								
T-AOC (mmol/gprot)	0.070	0.079	0.086	0.092	0.084	0.079	0.003	0.492
MDA (nmol/ml)	0.16	0.19	0.22	0.18	0.20	0.25	0.01	0.507
SOD (U/mgprot)	26.87	30.64	29.56	32.44	31.20	29.84	0.67	0.247
CAT (U/mgprot)	11.03	11.12	11.83	12.50	12.27	11.25	0.33	0.733
GSH-Px (U/mgprot)	79.88	78.87	71.99	94.93	96.21	81.71	3.56	0.283

*T-AOC, total antioxidant capacity; MDA, malondialdehyde; SOD, superoxide dismutase; CAT, catalase; GSH-Px, glutathione peroxidase*.

a, b, c *Different superscripts within a row indicate a significant difference (P <0.05)*.

1 *Con, basal diet; CTC, basal diet supplemented with 75 mg/kg chlortetracycline; LEO, basal diet supplemented with 100 mg/kg common EOs; HEO, basal diet supplemented with 500 mg/kg common EOs; LMEEO, basal diet supplemented with 100 mg/kg MEEOs; HMEEO, basal diet supplemented with 500 mg/kg MEEOs*.

2 *SEM means standard error of the means (n = 8)*.

### Relative mRNA Expressions of Pro-inflammatory Cytokines and Toll-Like Receptor in Small Intestine

As shown in Table 4, piglets fed 500 mg/kg common EOs (HEO group) exhibited significantly increased relative mRNA expression of *IL-8* in the duodenum and *IL-1*β in the ileum compared with the other groups (*P* < 0.05). The relative mRNA expression levels of *TNF-*α, *TLR4* and *TLR8* in the duodenum and ileum were also significantly elevated in the HEO group compared with the other groups (*P* < 0.05).

**Table 4:** Effect of common EOs and MEEOs on the relative mRNA expressions of pro-inflammatory cytokine and toll-like receptor in the duodenum, jejunum and ileum of weaning piglets.

**Item**	**Group^1^**						**SEM^2^**	***P*-Value**
	**Con**	**CTC**	**LEO**	**HEO**	**LMEEO**	**HMEEO**		
**Duodenum**								
*IL-1β*	1.00	1.11	0.79	1.61	0.66	0.96	0.12	0.263
*IL-6*	1.00	0.68	0.70	0.83	0.83	1.21	0.08	0.324
*IL-8*	1.00^b^	0.94^b^	0.63^b^	3.21^a^	0.77^b^	1.55^b^	0.23	0.005
*TNF-α*	1.00^b^	0.74^b^	0.80^b^	2.29^a^	0.47^b^	0.53^b^	0.14	<0.001
*TLR4*	1.00^b^	1.26^b^	0.53^b^	2.87^a^	0.88^b^	1.68^b^	0.19	0.004
*TLR8*	1.00^b^	0.63^b^	1.25^ab^	2.09^a^	0.57^b^	0.39^b^	0.16	0.017
**Jejunum**								
*IL-1β*	1.00^b^	1.25^b^	2.55^a^	1.14^b^	0.90^b^	0.28^b^	0.20	0.029
*IL-6*	1.00	0.66	0.79	0.82	0.74	1.06	0.07	0.591
*IL-8*	1.00	0.86	1.02	0.39	0.35	0.78	0.09	0.077
*TNF-α*	1.00	0.97	1.36	0.88	0.46	0.57	0.11	0.242
*TLR4*	1.00	1.06	1.67	0.97	0.57	0.42	0.13	0.101
*TLR8*	1.00	1.15	1.99	1.59	1.63	1.10	0.16	0.406
**Ileum**								
*IL-1β*	1.00^bc^	1.64^ab^	1.40^b^	2.61^a^	0.33^c^	0.62^bc^	0.17	<0.001
*IL-6*	1.00	0.88	0.97	1.44	1.02	1.20	0.06	0.073
*IL-8*	1.00	2.29	1.64	2.34	1.10	1.08	0.19	0.093
*TNF-α*	1.00^b^	2.27^b^	1.64^b^	4.66^a^	0.86^b^	1.02^b^	0.28	<0.001
*TLR4*	1.00^b^	2.35^b^	2.2^b^	5.60^a^	0.85^b^	0.98^b^	0.33	<0.001
*TLR8*	1.00^b^	2.99^b^	3.19^b^	15.55^a^	1.56^b^	1.36^b^	0.95	<0.001

*IL, interleukin; TNF-α, tumor necrosis factor-α; TLR, toll-like receptors*.

a, b, c *Values with different letters were significantly different (P <0.05)*.

1 *Con, basal diet; CTC, basal diet supplemented with 75 mg/kg chlortetracycline; LEO, basal diet supplemented with 100 mg/kg common EOs; HEO, basal diet supplemented with 500 mg/kg common EOs; LMEEO, basal diet supplemented with 100 mg/kg MEEOs; HMEEO, basal diet supplemented with 500 mg/kg MEEOs*.

2 *SEM means standard error of the means (n = 8)*.

### SCFAs in Colonic Digesta

As shown in Table 5, dietary supplementation with 500 mg/kg MEEOs increased the colonic acetate content of piglets compared with the CTC and LEO groups (*P* < 0.05). Dietary treatment had no effect on propionate, butyrate and valerate in the colon of piglets.

**Table 5:** Effect of common EOs and MEEO on SCFAs contents in the colon of weaning piglets.

**Item**	**Groups^1^**						**SEM^2^**	***P*-Value**
	**Con**	**CTC**	**LEO**	**HEO**	**LMEEO**	**HMEEO**		
Acetate (μg/g)	82.96^ab^	81.71^b^	82.56^b^	83.78^ab^	86.29^ab^	95.66^a^	1.36	0.042
Propionate(μg/g)	116.89	118.47	120.95	106.71	113.20	119.57	2.81	0.739
Butyrate (μg/g)	68.59	63.42	67.82	62.59	62.10	66.70	2.05	0.912
Valerate (μg/g)	21.02	18.41	16.69	22.05	19.42	16.75	0.87	0.361

a, b *Values with different letters were significantly different (P < 0.05)*.

1 *Con, basal diet; CTC, basal diet supplemented with 75 mg/kg chlortetracycline; LEO, basal diet supplemented with 100 mg/kg common EOs; HEO, basal diet supplemented with 500 mg/kg common EOs; LMEEO, basal diet supplemented with 100 mg/kg MEEOs; HMEEO, basal diet supplemented with 500 mg/kg MEEOs*.

2 *SEM means standard error of the means (n = 8)*.

### Microbiota Composition and Diversity in Cecum and Colon

A total of 94,500 (cecum) and 93,842 (colon) clean tags were obtained, and 866 operational taxonomic units (OTUs) for the cecum and 970 OTUs were clustered. As shown in Table 6, dietary supplementation with 500 mg/kg EOs and MEEOs (HEO and HMEEO groups) increased the microbial richness (the total number of observed microbial species) in the cecum of piglets compared with the CTC and LEO groups (*P* < 0.05). In addition, the cecal digesta of piglets in the HEO, LMEEO and HMEEO groups had a significantly higher Chao 1 index than those in the CTC and LEO groups (*P* < 0.05). The microbial richness and alpha diversity in the colon were unaffected by the dietary treatments.

**Table 6:** Effect of common EOs and MEEOs on the alpha diversity of microbiota in cecum and colon.

**Item**	**Groups^1^**						**SEM^2^**	***P*-Value**
	**Con**	**CTC**	**LEO**	**HEO**	**LMEEO**	**HMEEO**		
**Cecum**
Observed species	724.14^abc^	705.13^bc^	685.38^c^	762.13^a^	748.63^ab^	765.50^a^	8.30	0.015
Chao1	730.13^abc^	710.35^bc^	690.77^c^	767.05^ab^	754.87^ab^	771.04^a^	8.34	0.016
Shannon	5.95	6.09	6.09	6.51	6.30	6.26	0.08	0.385
Simpson	0.94	0.94	0.95	0.97	0.94	0.94	0.004	0.458
**Colon**
Observed species	829.29	844.33	837.5	792.38	847.63	830.63	7.44	0.291
Chao1	859.59	881.85	869.98	823.87	878.8	863.47	7.92	0.317
Shannon	7.00	7.03	7.01	6.89	6.95	6.76	0.05	0.604
Simpson	0.98	0.98	0.98	0.98	0.97	0.96	0.002	0.296

a, b, c *Different superscripts within a row indicate a significant difference (P <0.05)*.

1 *Con, basal diet; CTC, basal diet supplemented with 75 mg/kg chlortetracycline; LEO, basal diet supplemented with 100 mg/kg common EOs; HEO, basal diet supplemented with 500 mg/kg common EOs; LMEEO, basal diet supplemented with 100 mg/kg MEEOs; HMEEO, basal diet supplemented with 500 mg/kg MEEOs*.

2 *SEM means standard error of the means (n = 8)*.

**Figure 1:**
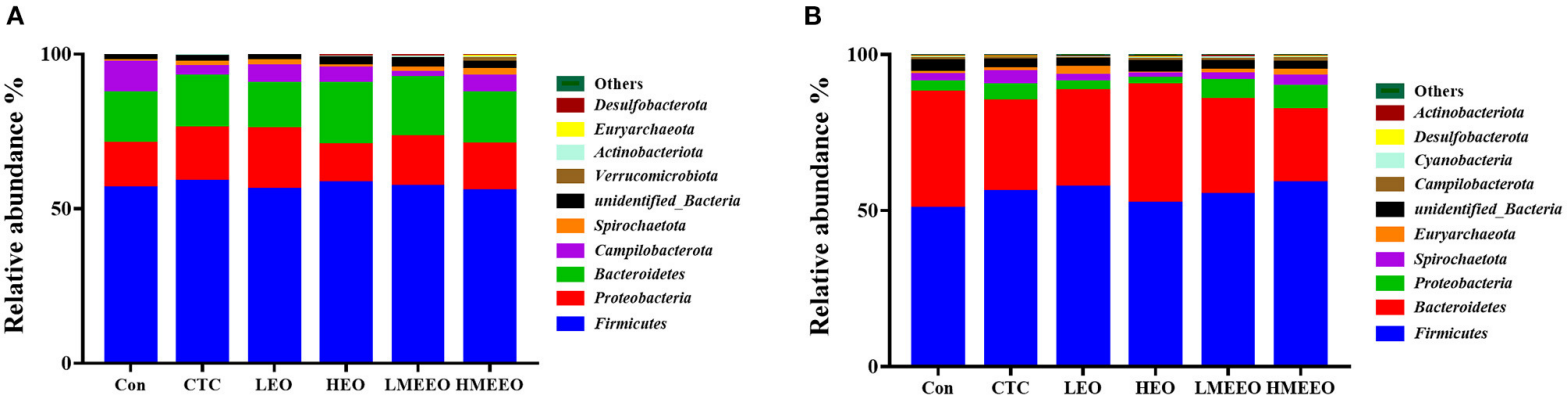
Effect of common EOs and MEEOs on taxonomic composition of the cecum and colon digesta at the phylum level. **(A)** Cecum, **(B)** Colon. Con, basal diet; CTC, basal diet supplemented with 75 mg/kg chlortetracycline; LEO, basal diet supplemented with 100 mg/kg common EOs; HEO, basal diet supplemented with 500 mg/kg common EOs; LMEEO, basal diet supplemented with 100 mg/kg MEEOs; HMEEO, basal diet supplemented with 500 mg/kg MEEOs.

**Figure 2:**
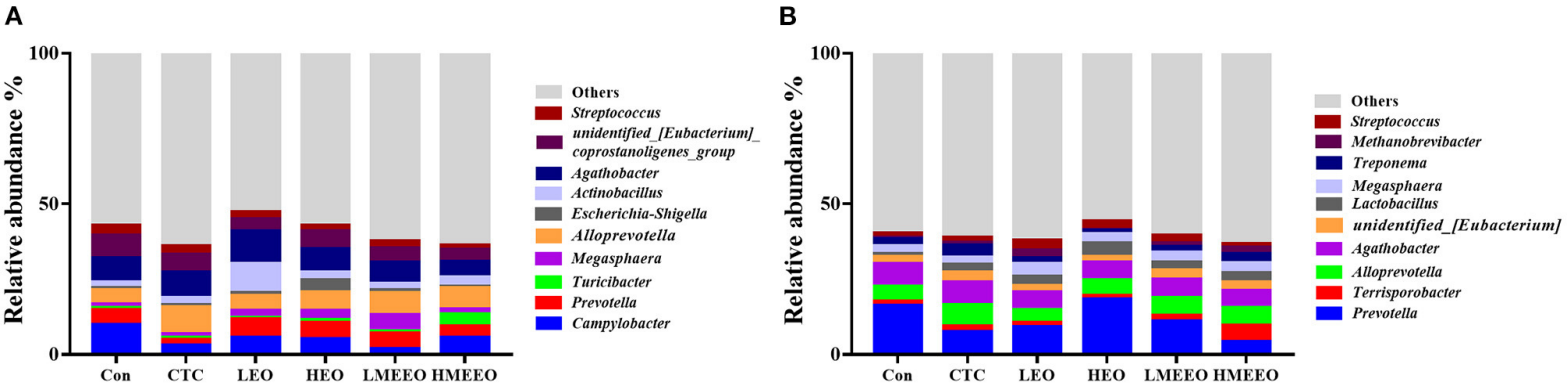
Effect of common EOs and MEEOs on taxonomic composition of the cecum and colon digesta at the genus level. **(A)** Cecum, **(B)** Colon. Con, basal diet; CTC, basal diet supplemented with 75 mg/kg chlortetracycline; LEO, basal diet supplemented with 100 mg/kg common EOs; HEO, basal diet supplemented with 500 mg/kg common EOs; LMEEO, basal diet supplemented with 100 mg/kg MEEOs; HMEEO, basal diet supplemented with 500 mg/kg MEEOs.

The intestinal microbial taxonomy was analyzed and is shown in Figures 1, 2. As shown in Figure 1, *Firmicutes* (55.49–58.43%), *Bacteroidetes* (14.67–19.71%), *Proteobacteria* (12.38–19.72%), *Campilobacterota* (1.69–9.89%), and *Spirochaetota* (0.45–2.13%) were the top 5 dominant phyla in the cecum. In the colon, *Firmicutes* (47.25–58.49%), *Bacteroidetes* (23.48–37.84%), *Proteobacteria* (2.39–5.09%), *Spirochaetota* (1.44–3.34%), and *Euryarchaeota* (0.33–2.67%) were detected. As shown in Figure 2, *Agathobacter, Actinobacillus, Alloprevotella, Prevotella, and Campylabacter* were the most dominant bacterial genera in the cecum while *Prevotella, Alloprevotella, Agathobacter, and Terrisporobacter* were the dominant bacterial genera in the colon.

**Figure 3:**
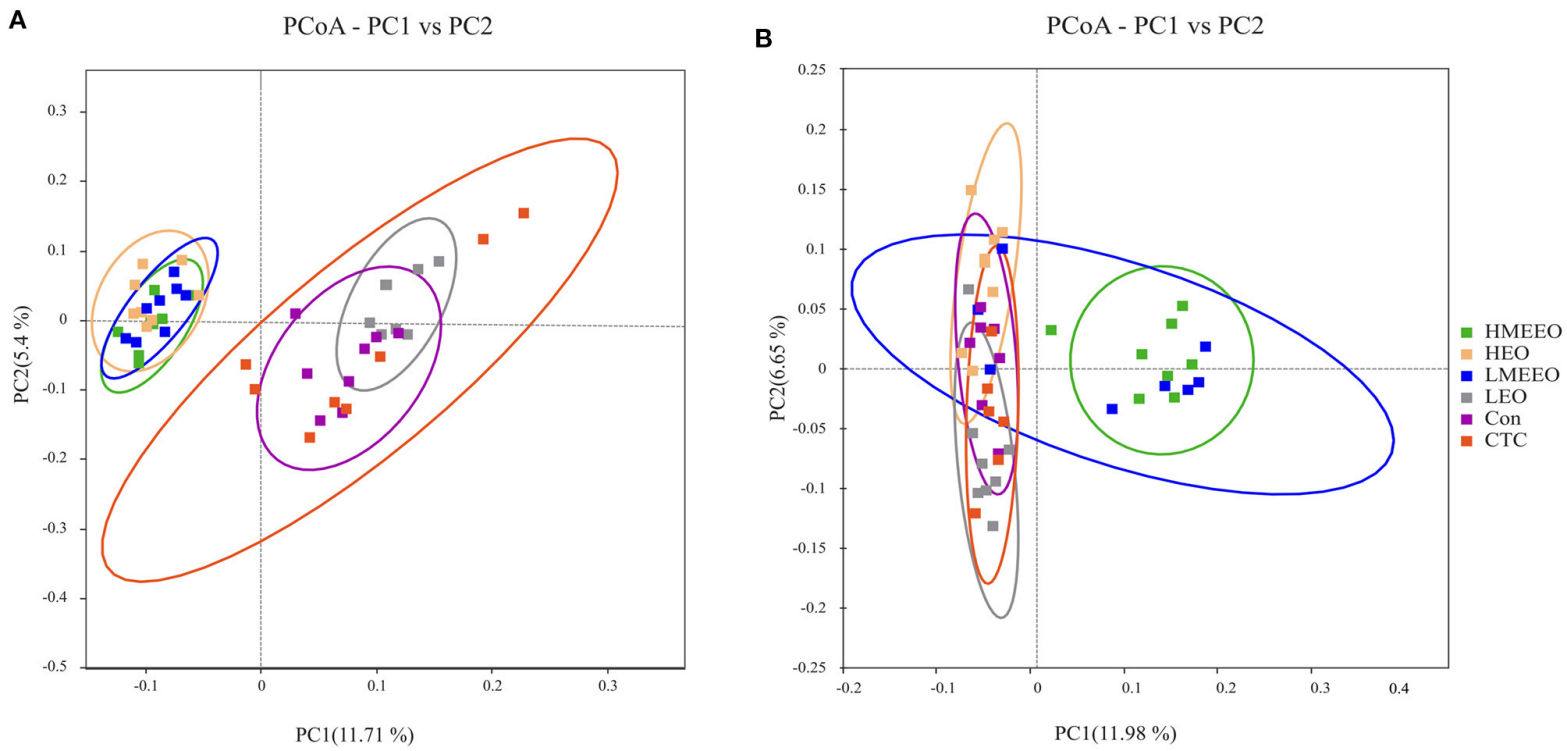
Effect of common EOs and MEEOs on microbiota beta diversity. **(A)** Principal Coordinates Analysis (PCoA) of cecum. **(B)** Principal Coordinates Analysis (PCoA) of colon. Con, basal diet; CTC, basal diet supplemented with 75 mg/kg chlortetracycline; LEO, basal diet supplemented with 100 mg/kg common EOs; HEO, basal diet supplemented with 500 mg/kg common EOs; LMEEO, basal diet supplemented with 100 mg/kg MEEOs; HMEEO, basal diet supplemented with 500 mg/kg MEEOs.

Based on the binary_jaccard distance, a principal coordinates analysis (PCoA) was performed to test whether the structure of the intestinal microbiota differed between the treatment groups. As shown in Figure 3, the cecum cecal microbiota communities in the HEO, LMEEO and HMEEO groups were significantly different from those in the Con, CTC and LEO groups. Similar to the PCoA results of the cecum, the colonic microbiota communities in the LMEEO and HMEEO groups were significantly different from those in the Con, CTC, LEO and HEO groups.

**Figure 4:**
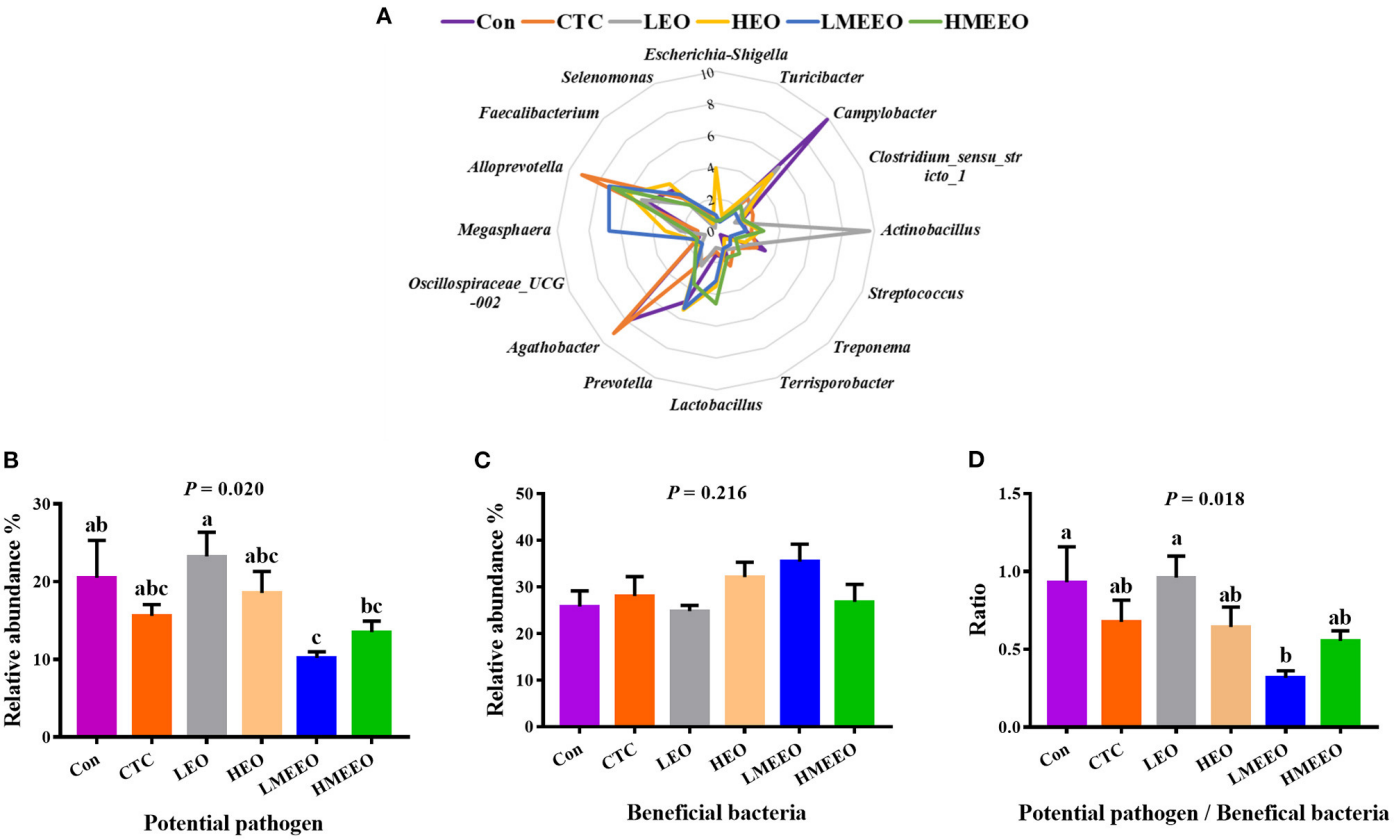
Effect of common EOs and MEEOs on the potential pathogens and beneficial bacteria in the cecum. **(A)** Radar graph of the relative abundance of top 8 potential pathogens and top 8 beneficial bacteria (%). The beneficial bacteria were presented on the left side of the graph, the potential pathogens were presented on the right side. **(B)** The sum of the relative abundance of the 8 potential pathogens in the treatment groups. **(C)** The sum of the relative abundance of the 8 potential pathogens in the treatment groups. **(D)** The ratio of 8 potential pathogens to 8 beneficial bacteria. Con, basal diet; CTC, basal diet supplemented with 75 mg/kg chlortetracycline; LEO, basal diet supplemented with 100 mg/kg common EOs; HEO, basal diet supplemented with 500 mg/kg common EOs; LMEEO, basal diet supplemented with 100 mg/kg MEEOs; HMEEO, basal diet supplemented with 500 mg/kg MEEOs. ^a, b, c^Values with different letters were significantly different (*P* < 0.05).

**Figure 5:**
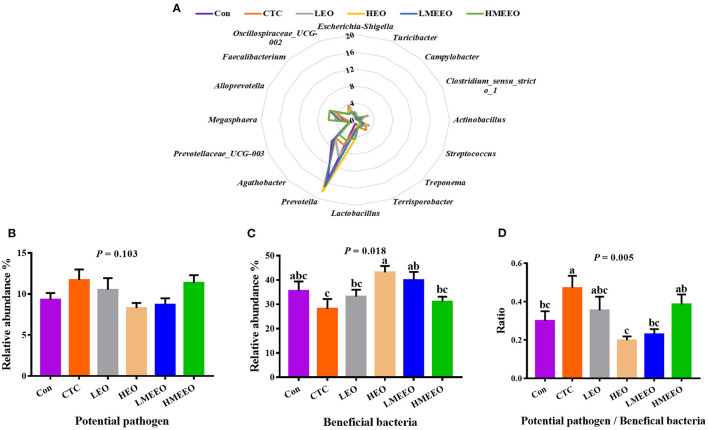
Effect of common EOs and MEEOs on the potential pathogens and beneficial bacteria in the colon. **(A)** Radar graph of the relative abundance of top 8 potential pathogens and top 8 beneficial bacteria (%). The beneficial bacteria were presented on the left side of the graph, the potential pathogens were presented on the right side. **(B)** The sum of the relative abundance of the 8 potential pathogens in the treatment groups. **(C)** The sum of the relative abundance of the 8 potential pathogens in the treatment groups. **(D)** The ratio of 8 potential pathogens to 8 beneficial bacteria. Con, basal diet; CTC, basal diet supplemented with 75 mg/kg chlortetracycline; LEO, basal diet supplemented with 100 mg/kg common EOs; HEO, basal diet supplemented with 500 mg/kg common EOs; LMEEO, basal diet supplemented with 100 mg/kg MEEOs; HMEEO, basal diet supplemented with 500 mg/kg MEEOs. ^a, b, c^Values with different letters were significantly different (*P* < 0.05).

To further analyze the effects of EOs and MEEOs on the pathogenic and probiotic bacteria, we selected and analyzed the 16 most abundant genera, which include 8 potential pathogens and 8 beneficial bacteria in the cecum and colon, respectively. Information on the selected potential pathogens and beneficial bacteria is listed in Supplementary Table 6. The differences in the relative abundance of these 16 genera between groups are shown in a radar graph (Figures 4A, 5A). Potential pathogens are presented on the right side of the graph, while beneficial bacteria are presented on the left side. In addition, the sum of the relative abundance of the 8 potential pathogens and the sum of the relative abundance of the 8 beneficial bacteria, as well as the ratio of potential pathogens to beneficial bacteria, were calculated, and the results are shown in Figures 4B–D, 5B–D.

For the cecum, Figure 4A (the radar graph) shows that the LMEEO, HMEEO and CTC groups had a higher percentage of beneficial bacteria (left side of the graph) than potential pathogens (right side of the graph) compared with the Con and LEO groups and the relative abundance of *Campylobacter* in the Con group and *Actinobacillus* in the LEO group were higher than those in the other groups. To more accurately determine the differences in the microbial composition, we compared the ratio of potential pathogens to beneficial bacteria between groups. The results (Figure 4) showed that the LMEEO group had the lowest abundance of potential pathogens (*P* < 0.05). The ratio of potential pathogens to beneficial bacteria in the LMEEO group was significantly lower than that in the Con and LEO groups (*P* < 0.05).

For the colon, Figure 5A shows that *Prevotella* was the dominant genus in the colon, especially for the HEO, Con, LMEEO and CTC groups. For most groups, the relative abundance of beneficial bacteria was higher than that of potential pathogens (*P* < 0.05). In addition, the ratio of potential pathogens to probiotic bacteria was significantly lower in the Con, HEO and LMEEO groups than in the CTC group (*P* < 0.05).

## Discussion

Thymol, carvacrol and cinnamaldehyde are unstable because they contain phenolic hydroxyl groups, hydroxyl groups and delocalized electron systems. These functional groups are sensitive to active oxygen species, light, environmental temperature and moisture. Thus, to reduce the damaging effect of the feed preparation process (high temperature) on the EOs, most feed manufacturers encapsulate the EOs prior to feed pelleting. In this study, we prepared a MEEO product and found that the degradation rate of the active ingredient in MEEOs was <10 % (in dry matter) after high-temperature exposure (Supplementary Figure 2), suggesting that the encapsulation treatment is greatly helpful in maintaining the stability of the EOs under high temperature.

The bioactive compounds in EOs have broad-spectrum antibacterial, antiviral, and antifungal activities and thus have been considered alternatives to antibiotic growth promoters and are often used as growth promotors for livestock (6). Li reported that dietary supplementation with 100 and 150 mg/kg MEEOs (the main active ingredients are thyme and cinnamaldehyde) improved the ADG and reduced the diarrhea index of weaning piglets (20). In this study, we found that dietary supplementation with dietary supplementation with 500 mg/kg MEEOs (HMEEO group) improved the ADG of weaned piglets and tended to promote feed intake, whereas piglets fed 500 mg/kg common EOs had the lowest ADG and ADFI during the first 2 weeks after weaning. This suggests that the addition of a high dose of common EOs may have affected feed intake due to unpleasant odor, leading to the reduced growth performance of piglets. Encapsulated EOs (MEEOs) are odor free, appetite and digestion stimulating (21), thus leading to an increase in ADFI and ADG. However, as the use of 500 mg/kg MEEOs was prolonged, the diarrhea in piglets was more severe than the use of 100 mg/kg common EOs and MEEOs, indicating that high doses of EOs and MEEOs are not suitable for long-term application in weaning piglets. On the other hand, piglets in the LMEEO group had the lowest serum DAO level. Diamine oxidase is distributed mainly in the villi of the small intestine, and it is often used as a plasma marker of intestinal mucosa damage (22). The present results suggested that 100 mg/kg MEEOs, rather than common EOs or 500 mg/kg MEEOs, can effectively protect the intestinal mucosa from weaning stress and prevent diarrhea. Overall, MEEOs were more effective than common EOs in improving piglet growth performance, and the addition of 100 mg/kg EOs and MEEOs to the diet was more effective than high doses.

Weaning stress in piglets is usually accompanied by oxidative stress in the intestinal tract. Intestinal oxidative stress often causes massive inflammation and enterocyte dysfunction and has adverse impacts on the growth performance and health of pigs (23). The phenolic hydroxyl groups in carvacrol and thymol have strong antioxidant activities (24), and EOs have been reported to facilitate the expression of antioxidant and detoxification enzymes (e.g., SOD, CAT, GSH-Px) through the Nrf2-ARE pathway, eliminate hydrogen peroxide and superoxide anions, and finally re-establish cellular redox homeostasis (25, 26). Tian reported that dietary supplementation with 100 mg/kg MEEOs (containing 4.5% cinnamaldehyde, 13.5% thymol and 82% feed grade carrier) enhanced the activities of catalase, total antioxidant capacity and glutathione peroxidase and reduced malondialdehyde levels in the small intestine of piglets (27). In this study, dietary supplementation with 100 mg/kg MEEOs increased the T-AOC and GSH-Px activity in the duodenum compared with the Con, CTC and LEO groups, and 500 mg/kg MEEOs increased jejunal GSH-Px activity compared with the Con and LMEEO groups, suggesting that MEEOs were more helpful in increasing the activities of antioxidant enzymes in the duodenum. MDA is the major product of lipid peroxidation, and it is often used as a biomarker of oxidative stress (28). Dietary supplementation with 500 mg/kg common EOs increased the MDA level in the duodenum compared with LEO, LMEEO and HMEEO group, suggesting that high dose of common EOs may cause oxidative stress in the duodenum, while the MEEOs may have avoided the instantaneous irritation of high dose of EOs to the duodenum because of the controlled release system (microencapsulation is a versatile technique to control the release of target) (29). Therefore, MEEOs would be more beneficial to improve the antioxidant capacity of the duodenum than common EOs, and 100 mg/kg MEEOs had the best effect.

Weaning stress is often accompanied by intestinal inflammation in piglets (30). We found that 500 mg/kg common EOs increased the relative mRNA abundance of *TLR4, TLR8* and *TNF-*α in the duodenum and ileum in the piglets. The relative mRNA abundance of *IL-8* in the duodenum and *IL-1*β in the ileum were also elevated after 500 mg/kg common EO administration. TLRs have been identified to play a significant role in the intestinal inflammatory process. *TLR8* was reported to mediate increased *IL-8* secretion from epithelial cells and inhibit the function of T regulatory cells, resulting in a reduction in immunosuppression and increased proinflammation in the gut (31). The generation of *TNF-*α and *IL-1*β may be enhanced by *TLR8* signaling and associated with mucosal inflammation (32). *TLR4* expression is correlated with *TNF-*α and *IL-6* mRNA levels. The expression of *TNF-?* was found to be elevated in the intestinal mucosa of ulcerative colitis patients, which can increase the expression of *TLR4* (33). Therefore, the use of a high dose of common EOs in the present study may have activated the inflammatory cytokine response, which led to the development of intestinal inflammation. Baldissera reported that thymol may promote inflammation and cytotoxicity by interfering with the hydrolysis of ATP and adenosine deamination in the extracellular environment and lead to a sustained proinflammatory deleterious cycle (34). Therefore, the addition of a high dose of unencapsulated EOs may have adverse effects on intestinal health. In contrast, encapsulating EOs enable the slower release of active ingredients, which may effectively prevent the intestine from being exposed to excessive EOs and causing inflammation.

Intestinal inflammation is often closely associated with an abnormal increase in intestinal pathogenic bacteria (35). EOs can regulate intestinal health by modulating intestinal microbiota, for example, by inhibiting the proliferation of pathogens. Thymol and carvacrol can integrate the outer membrane of targeted bacteria, leading to the depolarization of the cytoplasmic membrane of bacteria (36). In the present study, we analyzed the intestinal microbiota in the cecum and colon, which harbors the most diverse microbiome. The results showed that the observed species and Chao 1 index of cecum digesta were significantly higher in the LMEEO, HMEEO and HEO groups than in the CTC group. The Chao 1 index is a qualitative measure of alpha diversity, which reflects species richness. The higher the Chao 1 index is, the richer the number of species (37). Thus, it is possible to infer that EOs can increase cecum microbial diversity. In addition, the PCoA analysis showed that piglets fed MEEOs presented distinct PCoA clusters compared to those fed chlortetracycline, indicating that MEEOs regulated the gut microbiota in a manner different from that of chlortetracycline.

To further investigate the differences in the effects of EOs, MEEOs and chlortetracycline on the potential pathogens and the beneficial bacteria in the cecum and colon. We selected the top 16 genera in the cecum and colon for further investigation, the abundance of which accounted for approximately half of the total bacterial population and was therefore representative. Among the top 8 potential pathogens, *Escherichia-Shigella, Campylobacter, Treponema* and *Streptococcus* are the most familiar potential pathogens in the intestine, and they are frequently associated with intestinal inflammation, mucosal damage, diarrhea, inflammatory bowel disease and other intestinal diseases (38–41). *Turicibacter* is confirmed to be linked to host immunity and has been positively associated with intestinal inflammation (42). *Clostridium_sensu_stricto_1* has been reported to cause necrotizing enterocolitis in infants and piglets, which is a severe inflammation in the intestines that can cause death (43). *Actinobacillus* often colonizes the mucous membrane and causes inflammation, which can act as a reservoir for opportunistic infections (44). *Terrisporobacter*, an emerging anaerobic pathogen and acetobacterium, can cause surgical site infection (45). Among the top 8 beneficial bacteria, *Prevotella* is the most abundant genus in the colon of weaning piglets, and it is capable of metabolizing dietary fiber and producing SCFAs that are beneficial for intestinal health (46). *Agathobacter* is the most dominant genus in the cecum and can help to alleviate immune-related adverse events. A decrease in the abundance of this genus in the colon is associated with inflammatory bowel disease (IBD) (47, 48). *Lactobacillus* and *Megasphaera* are the dominant genera in the intestine of young animals and play an important role in maintaining intestinal health and improving the immune function of the body (49). Currently, some strains belonging to the two genera have been developed into probiotic products for regulating the function of the gastrointestinal tract (50, 51). *Alloprevotella* can produce SCFAs, mainly succinate and acetate, which could improve the intestinal epithelial barrier and prevent inflammation (52). *Faecalibacterium* provides energy to colonic epithelial cells and has the ability to promote the proliferation of probiotic bacteria, alleviate inflammation and maintain intestinal health (53). Oscillospiraceae_*UCG-002* has been found to be beneficial for piglets to obtain energy from plant-derived feed (54). The dominant cecum genus *Selenomonas* has been reported to possess phytase activity, which enables them to digest soluble sugars and lactic acid (55). The dominant colon genus *Prevotellacea*_*UCG-003* was the key bacterium in the intestinal microbiota of nondiarrheic piglets, suggesting that it is crucial for maintaining normal intestinal function (56). In the present study, the total relative abundance of the top 8 beneficial bacteria in the cecum and colon was higher than that of potential pathogens, which is important for maintaining intestinal health (57, 58). MEEOs (100 mg/kg) reduced the relative abundance of potential pathogens in the cecum and were more effective than common EOs (100 mg/kg), suggesting that MEEOs inhibited the proliferation of potential cecum pathogens better than common EOs. In the colon, compared with chlortetracycline, 100 mg/kg MEEOs significantly increased the relative abundance of beneficial bacteria and decreased the ratio of potential pathogens to beneficial bacteria, indicating that MEEOs can promote the proliferation of probiotic bacteria in the colon, while the use of CTCs was not conducive to the survival of probiotic bacteria in the colon; therefore, compared with CTCs, MEEOs were more conducive to the colonization of beneficial bacteria. On the other hand, 500 mg/kg MEEOs did not favor the colonization of beneficial bacteria in the colon, which may be related to the presence of high concentrations of EOs in the colon caused by the slow-release effect of microencapsulation, while high concentrations of EOs may have adverse effects on intestinal bacteria (59). For common EOs, degradation in the stomach and small intestine may result in the loss of most of the active groups, leading to the difficulty of full function (60).

The results from the intestinal microbiota analysis showed that most of the beneficial bacteria in the cecum and colon can regulate intestinal health by producing SCFAs. Thus, we further analyzed the content of short-chain fatty acids in the colon and found that 500 mg/kg MEEOs significantly increased the acetate level in the colonic digesta. Acetate can reach the peripheral circulation at relatively high amounts and then activate the parasympathetic nervous system, leading to increased ghrelin secretion and food intake (61). Therefore, the higher ADFI values of piglets in the HMEEO group may be related to the significant elevation of colonic acetate. However, the increased acetate in the colon may lead to diarrhea (62); therefore, the diarrhea of piglets in the HMEEO group was not effectively relieved, which may be related to the elevated colonic acetate.

## Conclusion

Collectively, our results demonstrated that MEEOs increased the ADG of piglets during the first 2 weeks after weaning, and improved intestinal antioxidative capacity in weaning piglets. The ratio of the relative abundance of potential pathogenic and beneficial bacteria in the colon of piglets was decreased after MEEOs treatment. Microencapsulation technology significantly reduced the adverse effect of EOs, such as the intestinal inflammation induced by high-dose EOs exposure. Chlortetracycline caused an increase in the ratio of the relative abundance of potential pathogenic and beneficial bacteria in the colon, which could potentially have adverse effects on the intestine. The addition of a high dose of MEEOs may have adverse effects on the intestine and could lead to diarrhea by increasing colonic acetate.

## Data Availability Statement

The datasets presented in this study can be found in Supplementary Material and online repositories (BioProject PRJNA777298, http://www.ncbi.nlm.nih.gov/bioproject/777298).

## Ethics Statement

The animal study was reviewed and approved by the Animal Care and Use Committee of the Institute of Subtropical Agriculture, Chinese Academy of Sciences.

## Author Contributions

XH and HN designed the experiments, revised the manuscript, and supervised the entire study. KM, JL, FL, and YX conducted experiments, collected, and analyzed the data. KM drafted the manuscript. All authors read and approved the final manuscript.

## Funding

This work was supported by the Guangdong Basic and Applied Basic Research Foundation (2019B1515210022).

## Conflict of Interest

The authors declare that the research was conducted in the absence of any commercial or financial relationships that could be construed as a potential conflict of interest.

## Publisher's Note

All claims expressed in this article are solely those of the authors and do not necessarily represent those of their affiliated organizations, or those of the publisher, the editors and the reviewers. Any product that may be evaluated in this article, or claim that may be made by its manufacturer, is not guaranteed or endorsed by the publisher.
