# Supplementary material for: Superiority of Microencapsulated Essential Oils Compared With Common Essential Oils and Antibiotics: Effects on the Intestinal Health and Gut Microbiota of Weaning Piglet
Source: Front Nutr. 2022 Jan 12;8:808106. doi: 10.3389/fnut.2021.808106 (PMC8790512; doi:10.3389/fnut.2021.808106)
Supplement: Supplementary file 1 [file Data_Sheet_1.docx]

***Supplementary Material***

**1 Supplementary Data**

**1.1 The morphology of MEEOs and EOs**

The morphology of MEEOs was analyzed through SEM and Laser Particle Size Analyzer. The results were shown in Supplementary Figure 1. The MEEOs are spherical in shape with a uniform size and are found as aggregated particles. Approximately 90% of the MEEOs have a size ranging from 100 μm to 1000 μm. The common EOs are irregularly shaped.


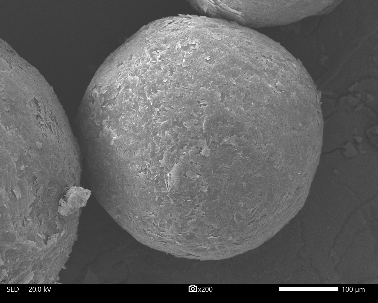

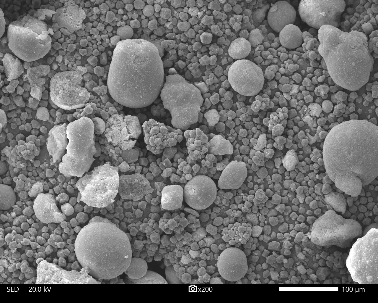


BA

AA

C

Supplementary Figure 1. The morphology of MEEOs. (A) The SEM images of MEEOs. (B) The SEM images of common EOs. (C) The particle size of MEEOs and common EOs.

**1.2 Thermal stability of MEEOs**

MEEOs were deposited at 40 °C for 6 months and 90°C for 3 minutes to evaluate the changes in its active gradients. The concentration of thymol, carvacrol and cinnamaldehyde in the MEEOs was determined according to previous study (2). Briefly, MEEO samples were ground and crushed, and then accurately weighed (200 mg ± 0.1mg) and transferred to 100 mL volumetric flask and made up the volume with methanol. After being thoroughly mixed, the mixture was filtrated through a 0.45 μm filter membrane, and the filtrate was collected for further analysis. The concentratins of carvacrol, cinnamaldehyde and thymol was determined by high performance liquid chromatograph (HPLC) using C18 column (4.6 mm×250 mm, 5.0 μm). The chromatographic separation was carried out using a mobile phase consisting of methanol, water and acetic acid (60:40:2, v/v/v). The column oven temperature was maintained at 30 °C, flow rate was set at 0.5 mL/min and the injection volume was 20 μL. The results are shown in Supplementary Figure 2. The retention of MEEOs was over 60 % when deposited at 40 °C for 6 months and over 90 % when deposited at 80 °C for 3 minutes.

Supplementary Figure 2. Thermal stability of MEEOs. (A) 40 °C for 6 months; (B) 80°C for 3 minutes.

**1.3 Microencapsulation yield of MEEOs**

Microencapsulation yield (MY) was determined according to Alvarez-Henao, M. V. e*t al*(1*)*. As shown in Supplementary Table 1, the MY of the MEEOs was 97.00 %-98.50 % in this study.

Supplementary Table 1. Microencapsulation yield of the MEEOs

| Item | 1 | 2 | 3 |
| --- | --- | --- | --- |
| MY | 97.1% | 97.0% | 98.5% |

**2 Supplementary Tables**

Supplementary Table 2. Composition and nutrient levels of the basal diet (as-fed basis, %).

| Ingredients | Content | Nutrient levels^2^ | Content |
| --- | --- | --- | --- |
| Corn | 59.90 | Digestible energy (Mcal / kg) | 14.72 |
| Soybean meal | 6.61 | Crude protein | 18.90 |
| Soybean protein concentrate | 8.00 | Calcium | 0.80 |
| Whey powder | 8.00 | Total protein | 0.52 |
| Fish meal | 5.00 | Available phosphorus | 0.33 |
| Soybean oil | 2.00 | Digestible lysine | 1.35 |
| Sucrose | 2.00 | Digestible methionine | 0.39 |
| Glucose | 3.75 | Digestible threonine | 0.79 |
| Limestone | 0.70 | Digestible tryptophan | 0.22 |
| Calcium hydrogen phosphate | 0.63 | Natrium | 0.36 |
| Choline chloride | 0.10 | Chlorine | 0.27 |
| Antioxidant | 0.05 |  |  |
| Citric acid | 0.90 |  |  |
| Table salt | 0.35 |  |  |
| Lysine (98%) | 0.59 |  |  |
| DL-Methionine | 0.09 |  |  |
| Threonine | 0.23 |  |  |
| Tryptophan (98%) | 0.05 |  |  |
| Premix^1^ | 1.00 |  |  |
| Dimethyl fumarate | 0.05 |  |  |
| Total | 100.00 |  |  |

^1^ The premix provided the following per kg of diets: VA_2_, 200 IU; VD_3 ,_ 220 IU; VE, 16 IU; VK_3_, 0.5 mg; VB_1_, 1 mg; VB_2_, 3.5 mg; VB_6_, 7 mg; VB_12_, 17.5 mg; biotin, 0.05 mg; folic acid, 0.3 mg; D-calcium pantothenate, 10 mg; nicotinic acid, 30 mg; Fe, 1120 mg; Cu, 85 mg; Mn, 60 mg; Zn, 320 mg; I, 0.1 mg; Se, 0.1 mg; Co, 0.1 mg.

^2^ Nutrient levels were all calculated values.

Supplementary Table 3. Primers used for quantitative PCR

| Target group | Sequence of primers (5′ to 3′) | GenBank accession No. |
| --- | --- | --- |
| *β-actin* | F: TGCGGGACATCAAGGAGAAG | XM_003124280.5 |
|  | R: AGTTGAAGGTGGTCTCGTGG |  |
| *IL-1β* | F: AGAGGGACATGGAGAAGCGA | [XM_021082463.1](https://www.ncbi.nlm.nih.gov/nucleotide/XM_021082463.1?report=genbank&log$=nucltop&blast_rank=3&RID=C67SGAA8013) |
|  | R: CCAGGAAGACGGGCTTTTGT |  |
| *IL-6* | F: TCTCTGGGATCATGGGGCA | [XM_021088726.1](https://www.ncbi.nlm.nih.gov/nucleotide/XM_021088726.1?report=genbank&log$=nucltop&blast_rank=1&RID=C67VJCMM013) |
|  | R: CGCCACCTCTGCCAGTTT |  |
| *IL-8* | F: TGAGAAGCAACAACAACAGCA | [AB057440.1](https://www.ncbi.nlm.nih.gov/nucleotide/AB057440.1?report=genbank&log$=nucltop&blast_rank=4&RID=C6866YVJ01R) |
|  | R: CAGCACAGGAATGAGGCATA |  |
| *TNF-α* | F: ACAGGCCAGCTCCCTCTTAT | [NM_214022.1](https://www.ncbi.nlm.nih.gov/nucleotide/NM_214022.1?report=genbank&log$=nucltop&blast_rank=9&RID=C66SER3T013) |
|  | R: CCTCGCCCTCCTGAATAAAT |  |
| *TLR4* | F: GCCATCGCTGCTAACATCATC | [NM_001293316.1](https://www.ncbi.nlm.nih.gov/nucleotide/NM_001293316.1?report=genbank&log$=nucltop&blast_rank=2&RID=C6848FZ2013) |
|  | R: CTCATACTCAAAGATACACCATCGG |  |
| *TLR8* | F: AGAGCTGCTAATTGGTGCCTT | [NM_214187.1](https://www.ncbi.nlm.nih.gov/nucleotide/NM_214187.1?report=genbank&log$=nucltop&blast_rank=7&RID=C684HMEE016) |
|  | R: AGGCAGGTCAGGAGCAAAAA |  |

Supplementary Table 4. Mass Spectrometry Operating Conditions

| Item | Value | Item | Value |
| --- | --- | --- | --- |
| Column temperature(℃) | 45.00 | Injection volume(μL) | 4.00 |
| Capillary (kV) | 2.70 | Source Temperature (℃) | 150.00 |
| Cone (V) | 30.00 | Desolvation Temperature (℃) | 450.00 |
| Cone Gas Flow (L/H) | 20.00 | Desolvation Gas Flow (L/H) | 1000.00 |

Supplementary Table 5. Liquid chromatography operating conditions

| Time(min) | Flow Rate（ml/min） | H_2_O_2_(containing 0.1% formic acid)(%) | Methanol(%) |
| --- | --- | --- | --- |
| 0.00 | 0.30 | 90.00 | 10.00 |
| 2.00 | 0.30 | 80.00 | 20.00 |
| 9.00 | 0.30 | 45.00 | 55.00 |
| 10.00 | 0.30 | 0.00 | 100.00 |
| 11.00 | 0.30 | 0.00 | 100.00 |
| 11.10 | 0.30 | 90.00 | 10.00 |
| 13.00 | 0.30 | 90.00 | 10.00 |

Supplementary Table 6. The information on the selected potential pathogens and beneficial bacteria

| Generic name | Classification | References |
| --- | --- | --- |
| *Escherichia-Shigella* | Potential pathogen | (3) |
| *Turicibacter* | Potential pathogen | (4) |
| *Campylobacter* | Potential pathogen | (5) |
| *Clostridium_sensu_stricto_1* | Potential pathogen | (6) |
| *Actinobacillus* | Potential pathogen | (7) |
| *Streptococcus* | Potential pathogen | (8) |
| *Treponema* | Potential pathogen | (9) |
| *Terrisporobacter* | Potential pathogen | (10) |
| *Lactobacillus* | Beneficial bacteria | (11) |
| *Prevotella* | Beneficial bacteria | (12) |
| *Agathobacter* | Beneficial bacteria | (13, 14) |
| *Megasphaera* | Beneficial bacteria | (15) |
| *Alloprevotella* | Beneficial bacteria | (16) |
| *Faecalibacterium* | Beneficial bacteria | (17, 18) |
| *Selenomonas* | Beneficial bacteria | (19, 20) |
| *Prevotellaceae_UCG-003* | Beneficial bacteria | (21) |
| *Oscillospiraceae_UCG-002* | Beneficial bacteria | (22) |

**References**

1. Alvarez-Henao MV, Saavedra N, Medina S, Cartagena CJ, Alzate LM and Londono-Londono J. Microencapsulation of lutein by spray-drying: Characterization and stability analyses to promote its use as a functional ingredient. *Food Chem* (2018) 256:181-187. doi:10.1016/j.foodchem.2018.02.059

2. Jiménez-Salcedo M and Tena MT. Determination of cinnamaldehyde, carvacrol and thymol in feedstuff additives by pressurized liquid extraction followed by gas chromatography–mass spectrometry. *Journal of Chromatography A* (2017) 1487:14-21. doi: 10.1016/j.chroma.2017.01.042

3. Croxen MA and Finlay BB. Molecular mechanisms of Escherichia coli pathogenicity. *Nature Reviews Microbiology* (2010) 8:26-38. doi:10.1038/nrmicro2265

4. Cuív PÓ, Klaassens ES, Durkin AS, Harkins DM, Foster L, McCorrison J*, et al*. Draft genome sequence of Turicibacter sanguinis PC909, isolated from human feces. *J Bacteriol* (2011) 193:1288-1289. doi:10.1128/JB.01328-10

5. Yang G, Yan Y, Zhang L, Ruan Z, Hu X, Zhang S and Li X. Porcine circovirus type 2 (PCV2) and Campylobacter infection induce diarrhea in piglets: Microbial dysbiosis and intestinal disorder. *Animal Nutrition* (2020) 6:362-371. doi: 1 0.1016/j.aninu.2020.05.003

6. Zhou J, He Z, Yang Y, Deng Y, Tringe SG and Alvarez-Cohen L. High-Throughput Metagenomic Technologies for Complex Microbial Community Analysis: Open and Closed Formats (2015) 6:e02288-14. doi:10.1128/mBio.02288-14

7. Samanta I and Bandyopadhyay S: Chapter 18 - Actinobacillus. In: *Antimicrobial Resistance in Agriculture*. Ed I. Samanta&S. Bandyopadhyay. Academic Press, (2020) doi: 10.1016/B978-0-12-815770-1.00018-3

8. Lun Z-R, Wang Q-P, Chen X-G, Li A-X and Zhu X-Q. Streptococcus suis: an emerging zoonotic pathogen. *The Lancet Infectious Diseases* (2007) 7:201-209. doi: 10.1016/S1473-3099(07)70001-4

9. Mamuad LL, Seo BJ, Faruk MSA, Espiritu HM, Jin SJ, Kim W-I*, et al*. Treponema spp., the dominant pathogen in the lesion of bovine digital dermatitis and its characterization in dairy cattle. *Veterinary Microbiology* (2020) 245:108696. doi: 10.1016/j.vetmic.2020.108696

10. Cheng MP, Domingo M-C, Lévesque S and Yansouni CP. A case report of a deep surgical site infection with Terrisporobacter glycolicus/T. Mayombei and review of the literature. *BMC Infectious Diseases* (2016) 16:529. doi:10.1186/s12879-016-1865-8

11. O’Callaghan J and O’Toole PW: Lactobacillus: Host–Microbe Relationships. In: *Between Pathogenicity and Commensalism*. Ed U. Dobrindt, J. H. Hacker&C. Svanborg. Springer Berlin Heidelberg, Berlin, Heidelberg (2013) doi:10.1007/82_2011_187

12. Iljazovic A, Amend L, Galvez EJC, de Oliveira R and Strowig T. Modulation of inflammatory responses by gastrointestinal Prevotella spp. – From associations to functional studies. *International Journal of Medical Microbiology* (2021) 311:151472. doi: 10.1016/j.ijmm.2021.151472

13. Hakozaki T, Richard C, Elkrief A, Hosomi Y, Benlaïfaoui M, Mimpen I*, et al*. The Gut Microbiome Associates with Immune Checkpoint Inhibition Outcomes in Patients with Advanced Non–Small Cell Lung Cancer. *Cancer Immunology Research* (2020) 8:1243. doi:10.1158/2326-6066.CIR-20-0196

14. Moustafa A, Li W, Anderson EL, Wong EHM, Dulai PS, Sandborn WJ*, et al*. Genetic risk, dysbiosis, and treatment stratification using host genome and gut microbiome in inflammatory bowel disease. *Clin Transl Gastroenterol* (2018) 9:e132-e132. doi:10.1038/ctg.2017.58

15. Bhute S, Pande P, Shetty SA, Shelar R, Mane S, Kumbhare SV*, et al*. Molecular Characterization and Meta-Analysis of Gut Microbial Communities Illustrate Enrichment of Prevotella and Megasphaera in Indian Subjects. *Front Microbiol* (2016) 7:660-660. doi:10.3389/fmicb.2016.00660

16. Li Y, Guo Y, Wen Z, Jiang X, Ma X and Han X. Weaning Stress Perturbs Gut Microbiome and Its Metabolic Profile in Piglets. *Scientific Reports* (2018) 8:18068. doi:10.1038/s41598-018-33649-8

17. Fenn K, Strandwitz P, Stewart EJ, Dimise E, Rubin S, Gurubacharya S*, et al*. Quinones are growth factors for the human gut microbiota. *Microbiome* (2017) 5:161. doi:10.1186/s40168-017-0380-5

18. Ferreira-Halder CV, Faria AVdS and Andrade SS. Action and function of Faecalibacterium prausnitzii in health and disease. *Best Practice & Research Clinical Gastroenterology* (2017) 31:643-648. doi: 10.1016/j.bpg.2017.09.011

19. Yanke LJ, Selinger LB and Cheng K-J. Phytase activity of Selenomonas ruminantium: a preliminary characterization (1999) 29:20-25. doi: 10.1046/j.1365-2672.1999.00568.x

20. Hespell RB, Paster BJ and Dewhirst FE: The Genus Selenomonas. In: *The Prokaryotes: Volume 4: Bacteria: Firmicutes, Cyanobacteria*. Ed M. Dworkin, S. Falkow, E. Rosenberg, K.-H. Schleifer&E. Stackebrandt. Springer US, New York, NY (2006) doi:10.1007/0-387-30744-3_33

21. Sun J, Du L, Li X, Zhong H, Ding Y, Liu Z and Ge L. Identification of the core bacteria in rectums of diarrheic and non-diarrheic piglets. *Scientific reports* (2019) 9:18675-18675. doi:10.1038/s41598-019-55328-y

22. Li H, Ma L, Li Z, Yin J, Tan B, Chen J*, et al*. Evolution of the Gut Microbiota and Its Fermentation Characteristics of Ningxiang Pigs at the Young Stage. *Animals (Basel)* (2021) 11:638. doi:10.3390/ani11030638
